# Determinants of child malnutrition and morbidity in Ethiopia: a structural equation modeling approach

**DOI:** 10.3389/fped.2025.1535957

**Published:** 2025-04-10

**Authors:** Birhanu Betela Warssamo, Denekew Bitew Belay, Ding-Geng Chen

**Affiliations:** ^1^ Department of Statistics, College of Natural and Computational Science, Hawassa University, Hawassa, Ethiopia; ^2^ Department of Statistics, Bahir Dar University, Bahir Dar, Ethiopia; ^3^ Department of Statistics, University of Pretoria, Pretoria, South Africa; ^4^ College of Health Solution, Arizona State University, Phoenix, AZ, United States

**Keywords:** under-five children, malnutrition, morbidity, anemia, structural equation modeling

## Abstract

**Background:**

Childhood malnutrition and morbidity remain significant public health challenges in Ethiopia, highlighting the need to assess the risk factors contributing to these issues for effective prevention and control strategies. Thus, this study aims to investigate the underlying risk factors by employing a structural equation model to analyze malnutrition as a mediator in the relationship between selected factors and morbidity.

**Methods:**

The study utilized data from the 2016 Ethiopia Demographic and Health Survey and a sample of 8,560 under-five children were considered. The structural equation model was used to examine the association between child malnutrition, morbidity, and potential risk factors. The structural equation model makes it possible to analyze malnutrition as a mediator of the association between selected risk factors and morbidity.

**Results:**

Out of the 8,560 sampled children, 12.80% were wasted, 34.75% were stunted, 23.91% were underweight, 13.9% had fever, 11.2% had diarrhea, and 59.7% had anemia. Birth interval, wealth index household, place of delivery, size of child at birth, number of children, and socioeconomic condition had a significant direct effect on childhood malnutrition and morbidity. The time to get water, toilet facility, and child is a twin variables had direct effects on childhood malnutrition and had no significant direct effects on childhood morbidity. Time to get water, birth interval, toilet facility, wealth index of household, child is a twin, place of delivery, size of child, and number of children exhibited an indirect effect on morbidity through malnutrition.

**Conclusions:**

The study revealed that there was a high prevalence of malnutrition and morbidity among under-five children in Ethiopia. Time to get water in min, place of delivery, size of child, and number of children showed a significant indirect and total effect on morbidity through malnutrition and socioeconomic conditions showed a significant total effect on morbidity via malnutrition. Implementing and extending programs such as community-based nutrition interventions for early childhood is critical, as early malnutrition showed long-term effects on growth and immunity, particularly in the regions of Affar, Dire Dawa, Gambela, Harari, Amhara, and Somali.

## 1 Background

Malnutrition, in all its forms, includes undernutrition (wasting, stunting, and underweight), and results from inadequate intake, imbalanced diets, or excessive nutrient loss. In early life, it increases the risk of infections, morbidity, and mortality, and impairs cognitive development (1). Morbidity in children can be caused by infectious disorders such as diarrhea, fever, cough, pneumonia, and tetanus, as well as chronic conditions such as congenital abnormalities and thalassemia (2). Child malnutrition is directly related to childhood morbidity, and malnutrition and morbidity are both linked to higher childhood mortality (3). Severe anemia is a major contributor to under-five mortality, significantly increasing morbidity and fatality rates in young children (4). Childhood morbidity indicates poor socioeconomic conditions and serves as a composite index reflecting a community's environment, economy, healthcare, and social norms (5). Higher-income households can afford better healthcare, housing, and sanitation, leading to improved health outcomes (6). In contrast, low socioeconomic status characterized by poverty, low family wealth, and limited parental education is a common risk factor for malnutrition and morbidity (7).

Malnutrition-related causes led to 5.2 million deaths among children under 5 years old, accounting for over half of all child deaths (1). Over 80% of the world's children live in resource-limited countries, where childhood mortality and morbidity rates are highest (4). Each year, approximately 14 million children under 5 die from hunger and disease, which is 40,000 per day or 2,000 per hour, 98% of whom are in low-income countries (8). Diarrhea, fever, cough, and malaria are the primary causes of death in these countries, over 60% of which could be prevented at a reasonable cost (9). Alarmingly, 15,000 children die daily from treatable diseases such as diarrhea, fever, and malaria (10). Globally, almost 149 million children were stunted in 2019, with 50 million wasting (11). In 2016, an estimated 159 million under-five children were stunted, representing 23.8% of the global total, a 15.8% decrease from 255 million in 1990 (12). In 2020, Asia accounted for over half of all stunted and wasted under-five children, while Africa accounted for two out of every five stunted and more than one-quarter of all wasted children (13). The World Health Organization (WHO) Global Nutrition Target (GTN) of reducing stunting by 40% and wasting below 5% by 2025 and the Sustainable Development Goal of ending all forms of malnutrition by 2030, appear unrealistic (14), and several African countries are still far from meeting the ambitious WHO GTN to reduce the number of stunted and wasted children (4). For example, out of Africa's 59 million stunted and 14 million wasted children in 2019, the East African area accounted for 41% of all stunting and 29% of all wasting (14). Sub-Saharan Africa and Southern and Central Asia accounted for more than half (2.8 million) and nearly one-third (1.5 million) of all child deaths, respectively (15). Countries such as Nigeria, India, Pakistan, the Democratic Republic of the Congo, and Ethiopia accounted for nearly half (49%) of all under-five fatalities in 2019 (16). In rural Ethiopia, 49.7% had diarrhea, 40.9% had fevers, and 38.0% had cough episodes (17). Malnutrition, particularly among women and children, is caused by a number of interconnected factors, including food insecurity, inadequate healthcare, poor maternal education, and a lack of access to clean water and sanitation (18–21). Childhood morbidity and mortality in Ethiopia remain high due to the burden of highly prevalent diseases such as diarrhea, fever, cough, malaria, and human immunodeficiency virus (HIV)–acquired immunodeficiency syndrome (AIDS), of which only diarrhea accounts for more than one in every 10 (13%) child deaths in Ethiopia (10). Ethiopia has one of the highest under-five child mortality rates, with an annual rate decrease of 4.7% (12). In 2019, the country had an average under-five mortality rate of 51 deaths per 1,000 live births (22). Diseases such as acute respiratory infection (ARI), fever, and diarrhea are among the leading causes of under-five mortality in Ethiopia (17). Every year, more than 2 million under-five children in the world's poorest communities die as a result of diarrhea (23).

National programs such as the Productive Safety Net Program (PSNP) and the Health Extension Program (HEP) seek to relieve food insecurity and enhance healthcare access in Ethiopia, but their scope and effectiveness differ by region (24). Childhood malnutrition and morbidity metrics are inherently unobservable as a single variable. The complex relationships between stunting, wasting, underweight, diarrhea, fever, and anemia, as well as their associations with numerous risk factors, remain poorly understood. However, recognizing these connections is critical for creating tailored and successful interventions. Despite the urgent need, few research studies have thoroughly investigated the underlying causes of childhood malnutrition and morbidity in Ethiopia. Identifying these major determinants could pave the way for more effective treatments to reduce childhood mortality and enhance overall health outcomes in the country. Therefore, this study aimed to identify common determinants of childhood malnutrition and morbidity using structural equation models.

## 2 Methods and materials

### 2.1 Study setting, design, and data source

**Figure 1:**
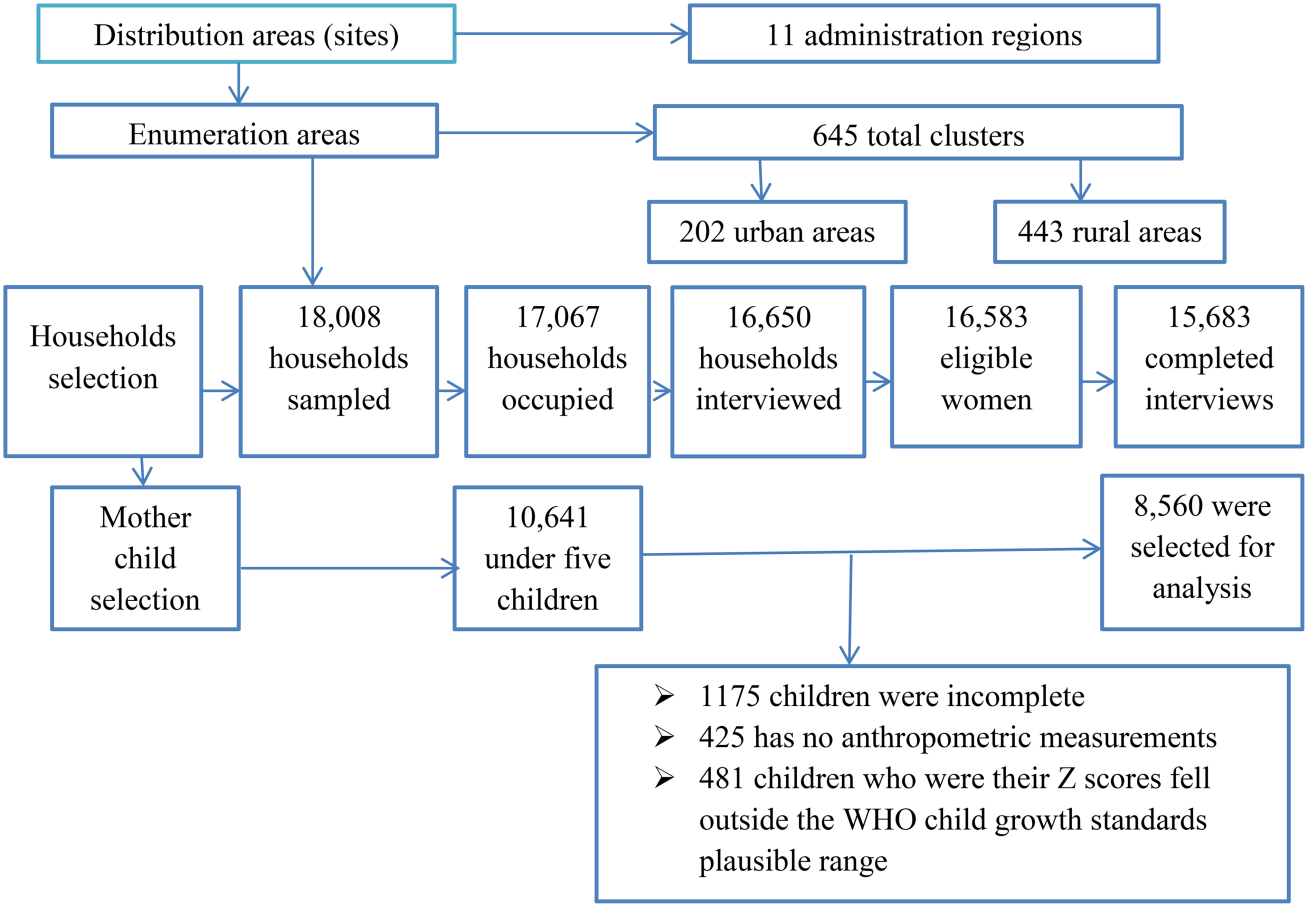
Representation of mother–child pair selection from EDHS data (2016).

The 2016 Ethiopia Demographic and Health Survey (EDHS) was used in this study. This was Ethiopia's fourth Demographic and Health Survey (DHS), following those conducted in 2000, 2005, and 2011 (17). It was carried out by the Central Statistical Agency (CSA) and Inner City Fund (ICF) International from 18 January to 27 June 2016 (25). The survey used a two-stage cluster sampling design that stratified regions into urban and rural areas. A two-stage cluster sampling design with urban and rural regional strata yielded 21 sample strata and a total of 645 clusters were studied, comprising 202 clusters in cities and 443 in rural areas. The final sample consisted of 15,683 households, with 5,348 from urban and 10,335 from rural areas. Data from these families were gathered and analyzed for 8,592 children under the age of 5 years who met the study's inclusion criteria of being under 5 years old in Ethiopia. However, those who were children aged 5 years or older and those whose mothers were not included in the household questionnaire or interviewed were excluded. The entire EDHS report (25) provides more information on the methods of the survey and the nutritional status measures and details of the sampling procedure are explained in Figure 1.

#### 2.1.1 Malnutrition measures

A child was considered stunted, underweight, or wasted if their height-for-age z-score (HAZ), weight-for-age z-score (WAZ), or weight-for-height z-score (WHZ) was more than 2 standard deviations below the WHO's growth reference levels for a healthy population (12). Stunting suggests chronic undernutrition in children; wasting indicates acute undernutrition; and being underweight indicates both chronic and acute undernutrition (26). Inadequate growth throughout childhood can result in poor health outcomes and an increased risk of premature death in adulthood (27).

### 2.2 Structural equation modeling

The use of structural equation modeling (SEM) entails several processes, including the construction of the theoretical conceptual model, the specification of the mathematical model, the determination of the model's evidence, the determination of the model fit, and the evaluation of the model's goodness of fit. SEM allows for the testing of research hypotheses in a single method by modeling complex interactions between numerous observable and latent variables. Based on theory, previous empirical findings, or both, the researcher develops hypotheses about the relationships between variables. They can be either direct or indirect, with intervening influences mediating the effect of one variable on another (28).

**Figure 2:**
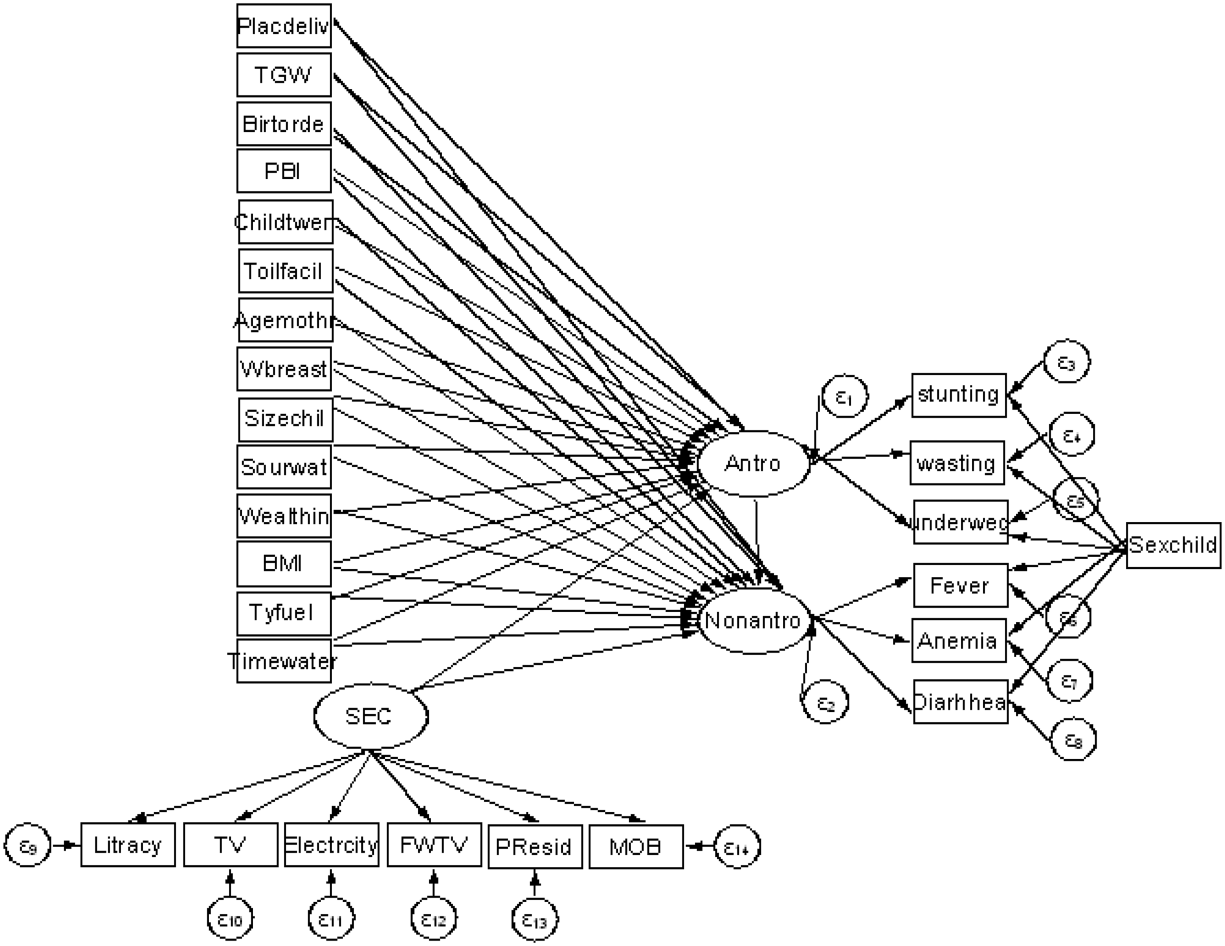
Proposed theoretical SEM path diagram of child malnutrition, socioeconomic condition, and morbidity in Ethiopia.

Figure 2 shows the multiple indicators and multiple causes (MIMIC) model, which was adopted from Chen and Yung (28). In this model, malnutrition, socioeconomic condition, and morbidity are latent variables. We can conceptualize the measured variables, namely, stunting, wasting, and underweight, as being the realization of childhood malnutrition. Diarrhea, anemia, and fever are the realization of childhood morbidity, and literacy, availability of television, electricity, frequency of watching television, and place of residence are the realization of socioeconomic condition. This can be quantified using the latent variables of malnutrition, socioeconomic condition, and morbidity. Arrows denote the direction of influence and oval shapes denote latent variables generated by the model whereas rectangles denote observed variables. Finally, the circles represent the error term. The MIMIC model consists of two parts: the measurement model, which defines the relations between the three latent variables (i.e., malnutrition, morbidity, and socioeconomic conditions) and its observed variables (indicators), and the structural model, which displays the causal links among exogenous and endogenous variables. The literacy, availability of television, electricity, mobile phone ownership, frequency of watching TV, and place of residence variables are indicative of the latent variable “socioeconomic condition” as they capture key dimensions of socioeconomic status, including access to education, technology, and essential infrastructure.

### 2.3 Model diagnostics

In structural equation modeling, it is recommended that various goodness-of-fit criteria be used in conjunction with overall fit measurements (29). As a result, the measurement indices range from poor fit to perfect fit, and various structural equation modeling programs report a range of the most common model fits as follows:

Cronbach's alpha: $\alpha =\frac{k}{k-1}\left(1-\frac{\sum \sigma_{i}^{2}}{\sigma_{t}^{2}}\right)$ where a higher value (typically above 0.7) indicates acceptable reliability. *k* is the number of items (indicators), $\sigma_{i}^{2}$ is the variance of each individual item, and $\sigma_{t}^{2}$ is the total variance of the sum of all items.

Average variance extracted (AVE): $\mathrm{AVE}=\frac{\sum \lambda_{i}^{2}}{\sum \lambda_{i}^{2}+\sum \varepsilon_{i}}$, where an AVE value of 0.50 or higher is considered good. $\lambda^{2}$ is the squared factor loading for each indicator and $\varepsilon_{i}$ is the error variance of indicator *i*.

Composite reliability (CR): $\mathrm{CR}=\frac{{\left(\sum \lambda_{i}\right)}^{2}}{{\left(\sum \lambda_{i}\right)}^{2}+\sum \varepsilon_{i}}$, where, if CR >0.70**,** the construct is considered reliable. *λ*_i_ is the standardized factor loadings of observed indicators, and$\varepsilon_{i}$ is the error variance of indicator i, and is calculated as $1-\lambda_{i}^{2}$.

Comparative fit index (CFI): $\mathrm{CFI}=1-\frac{\chi_{\mathrm{mod}el}^{2}-df_{\mathrm{model}}}{\chi_{\mathrm{null}}^{2}-df_{\mathrm{null}}}$, where values above 0.90 or 0.95 generally indicate a good fit.

Standardized root mean square residual (SRMR): $\mathrm{SRMR}=\sqrt{\frac{1}{P(P+1)}}\sum_{i\neq j}{\left(S_{ij}-\sum_{ij}\right)}^{2}$, where *P* is the number of observed variables in the model, $S_{ij}$ is the observed covariance (or correlation) between variables *i* and *j* in the dataset, and $\sum_{ij}$ is the model-implied covariance (or correlation) between variables *i* and *j*. SRMR values below 0.08 are generally considered acceptable.

Tucker–Lewis index (TLI) or non-normed fit index (NNFI): $\mathrm{TLI}=\frac{\frac{\chi_{\mathrm{null}}^{2}}{df_{\mathrm{null}}}-\frac{\chi_{\mathrm{model}}^{2}}{df_{\mathrm{model}}}}{\frac{\chi_{\mathrm{null}}^{2}}{df_{\mathrm{null}}}-1}$, where $\chi_{\mathrm{null}}^{2}$ is the chi-square statistic, $\chi_{\mathrm{model}}^{2}$ is the chi-square statistic for the proposed model. $df_{\mathrm{null}}$ is the degrees of freedom for the null model and $df_{\mathrm{model}}$ is degrees of freedom for the proposed model. TLI values above 0.90 or 0.95 suggest a good fit.

These goodness-of-fit criteria are based on differences in variance–covariance matrices between observed (original) and model-implied (replicate) (30).

## 3 Results

### 3.1 Descriptive statistics results

The percentage distribution of childhood malnutrition and morbidity is shown in Table 1. As indicated, the prevalence of malnutrition and morbidity was more common among children who were living in rural areas in comparison to children living in urban areas. In total, 82.8%, 81.6%, 83%, 88.4%, and 85.5% of the children living in rural areas had diarrhea, fever, or anemia, or were stunted, wasted, or underweight, respectively. Across all health indicators, the children in rural areas were disproportionately affected compared to their urban counterparts. These results underscore the disparity in health and nutritional status between urban and rural areas in Ethiopia. The distribution of child health indicators across maternal age groups at first birth indicates that the highest percentages of stunted (50.9%), wasted (49.7%), and underweight children (51.0%) were associated with mothers who gave birth between the ages of 15 and 19 years. Similarly, the highest prevalence of diarrhea (50.2%), fever (50.1%), and anemia (49.2%) were associated with mothers who gave birth between the ages of 15 and 19 years. Younger maternal age at first birth, particularly under 20 years, was associated with poorer health outcomes in children, including higher rates of malnutrition (stunting, wasting, and underweight) and higher morbidity (diarrhea, fever, and anemia). These patterns may reflect the challenges younger mothers face. The distribution of health indicators by the sex of the child indicates that the proportion of stunted (53.4%), wasted (55.9%), and underweight (55.9%), and the prevalence of diarrhea (52.8%), fever (51.5%), and anemia (50.7%) were slightly higher among boys than girls. The results suggest a marginally higher prevalence of malnutrition- and morbidity-related indicators among male children compared to female children. These differences could be influenced by several factors, including biological differences in metabolism and immune responses, and potential gender-based variations in care practices. The distribution of childhood health indicators based on the respondent's employment status (full-time, part-time, or irregular) indicates that children with part-time employed mothers have the highest prevalence of stunting (70.3%), wasting (70.4%), underweight (72.9%), diarrhea (63.1%), fever (64.9%), and anemia (64.9%). Children of mothers with part-time employment face the highest prevalence across all malnutrition and morbidity indicators, followed by children of mothers with full-time employment, while children of irregularly employed mothers exhibited lower rates overall. The majority of stunted (90.6%), wasted (83.9%), and underweight (89.5%) children, and those with fever (86.7%), diarrhea (87.6%), and anemia (88.4%) were exclusively breastfed, indicating that malnutrition and morbidity in these groups may be influenced by factors beyond breastfeeding, such as maternal nutrition, household socioeconomic conditions, or long-term food security. Stunting (49.7%), wasting (53.7%), underweight (51.4%), fever (54.9%), diarrhea (53.1%), and anemia (52.8%) were more prevalent among children in households where accessing water takes 30 min or more and stunting(6.8%), wasting (5.8%), underweight (7.0%), fever (54.9%), diarrhea (7.5%), and anemia (6.8%) were less prevalent in households where accessing water takes less than 5 min. Across all indicators, i.e., stunting, wasting, underweight, diarrhea, fever, and anemia, the prevalence of malnutrition and morbidity were highest in households where accessing water takes 30 min or more. The time burden of water collection may limit mothers’ capacity to provide adequate nutrition, maintain hygiene, and ensure safe water usage, all of which are crucial for preventing malnutrition and infection in children. Children in male-headed households exhibited higher rates of malnutrition [stunting (79.7%), wasting (76.5%), and underweight (76.5%)] and morbidity [diarrhea (81.8%), fever (78.5%), and anemia (78.8%)] compared to children in female-headed households. Female-headed households may adopt different caregiving practices, prioritization of child nutrition, or healthcare-seeking behaviors that positively influence child health outcomes. Across all indicators, children of mothers with no formal education exhibit the highest rates of malnutrition [stunting (71.6%), wasting (72.6%), and underweight (76.4%)] and morbidity [diarrhea (61.2%), fever (61.3%), and anemia (65.9%)]. These findings strongly suggest that maternal education has a protective effect on child health. Mothers with primary or higher education levels are likely better equipped with knowledge of nutrition, hygiene, and healthcare practices, which positively affects their children's health outcomes. The children who were placed at the breast within an hour after birth had greater rates of stunting (83.8%), wasting (84.7%), underweight (83.9%), diarrhea (82.0%), fever (80.2%), and anemia (84.3%), indicating that delaying breastfeeding was related with poorer health outcomes. Children breastfed within the first hour had significantly lower rates of all health concerns, demonstrating that early breastfeeding promotes better health outcomes. Children who are breastfed promptly have better morbidity outcomes (lower rates of diarrhea, fever, and anemia), indicating that immediate breastfeeding offers health benefits, particularly against infections and nutritional deficits. This pattern stresses the significance of starting breastfeeding early, ideally right after birth, to improve child health and reduce the risk of malnutrition and morbidity (Table 1).

**Table 1:** Percentage distribution of childhood malnutrition and morbidity (EDHS, 2016).

Variable	Stunted	Wasted	Underweight	Had diarrhea	Had fever	Had anemia
Type of place of residence						
Urban	11.6	14.4	9.7	17.2	18.4	17.0
Rural	88.4	85.6	90.3	82.8	81.6	83.0
Sex of household head						
Male	79.7	76.5	78.3	81.8	78.5	78.8
Female	20.3	23.5	21.7	18.2	21.5	21.2
Sex of child						
Male	53.4	55.9	54.1	52.8	51.5	50.7
Female	46.6	44.1	45.9	47.2	48.9	49.3
Given child anything other than breast milk						
No	90.6	83.9	89.5	87.6	86.7	88.4
Yes	9.4	16.1	10.5	12.4	13.3	11.6
Respondent employed all year/seasonal						
Full-time throughout the year	24.0	25.8	22.6	30.3	27.5	29.1
Part-time or only during certain seasons	70.3	70.4	72.9	63.1	64.9	66.6
Irregular	5.7	3.8	4.5	6.5	7.6	4.3
Father’s age group						
24 and younger	22.3	27.0	22.2	26.8	28.4	25.4
25–34	52.1	49.7	52.8	53.2	49.7	52.7
35–44	23.3	21.8	22.9	18.6	20.6	20.5
45 and older	2.3	1.5	2.1	1.3	1.3
Number of children under 5 in the household						
Below 1	32.9	28.3	29.3	41.3	41.9	32.3
2–3	64.5	67.9	67.8	56.7	54.9	64.5
4 and above	2.6	3.8	2.9	2.0	3.3	3.2
Time to get water resources in min						
Below 5	6.8	5.8	7.0	7.5	7.0	6.8
6–16	17.6	16.4	15.6	15.0	15.2	15.8
16–30	25.9	24.1	26.0	24.4	22.9	24.6
30 and above	49.7	53.7	51.4	53.1	54.9	52.8
Age of mother at first birth						
Under 15	14.6	15.2	15.3	12.8	13.3	13.5
15–19	50.9	49.7	51.0	50.2	50.1	49.2
20–24	27.6	28.0	27.3	29.8	29.2	29.2
25 and older	7.0	7.1	6.4	7.1	7.4	8.1
Interval of marriage to first birth in months						
Less than 12	26.2	28.6	27.6	25.9	25.9	26.9
12–24	33.0	31.6	32.3	31.4	31.7	33.5
25 and more	40.8	39.7	40.2	42.8	42.4	39.6
Religion						
Christian	47.4	37.3	41.9	54.5	55.1	42.7
Muslim	51.9	61.6	57.4	44.9	44.2	56.5
Other	0.7	0.6	0.7	0.6	0.7	0.8
When was the child put to breast?						
After an hour	83.8	84.7	83.9	82.0	80.2	84.3
Within the first hour	5.0	4.8	5.0	4.7	4.1	4.9
Immediately	11.2	10.5	11.1	13.4	15.7	10.8
Multiple births						
Single	97.0	97.5	96.8	97.7	97.2	97.6
Multiple	3.0	2.5	3.2	2.3	13.9	2.4
Duration of breastfeeding						
Not breastfeeding	3.7	3.6	4.0	2.1	2.6	3.5
Breastfeeding	96.3	96.4	96.0	97.9	97.4	96.5
Place of delivery						
Home	76.3	75.5	80.3	63.3	63.3	68.3
Health facility	23.7	24.5	19.7	36.7	36.7	31.7
Size of child at birth						
Small	32.1	37.8	35.9	34.7	33.9	30.4
Average	41.9	39.0	40.8	36.1	35.9	40.9
Large	26.1	23.2	23.3	29.2	30.2	28.7
Source of drinking water						
Public tap	17.2	16.9	16.9	15.8	16.1	16.2
Protected spring	8.6	7.7	8.9	9.2	8.4	7.4
Other	74.1	75.4	74.2	74.9	75.4	76.3
Birth order of the child						
First	17.7	17.6	16.2	20.5	21.6	19.2
Second	15.5	14.0	14.5	15.8	16.7	16.1
Third or later	66.7	68.4	69.3	63.7	61.7	64.7
Preceding birth interval in months						
Less than 24	25.6	25.4	27.7	20.4	20.1	23.2
25–48	53.7	55.5	56.0	46.6	51.2	52.4
49 and above	20.8	19.2	16.3	33.0	28.7	24.4
Literacy						
Cannot read at all	81.2	80.2	84.9	72.4	73.0	76.1
Can read	18.8	19.8	15.1	27.6	27.0	23.9
Type of toilet facility						
Flush toilet	1.8	2.8	1.5	3.6	4.2	3.4
Latrine	48.4	40.6	43.9	53.2	49.5	48.5
No facility	49.7	56.5	54.6	43.2	46.4	48.1
Type of cooking fuel						
Electricity	1.9	2.1	1.4	4.0	5.4	4.1
Traditional fuel	98.1	97.9	98.6	96.0	94.6	95.9
Wealth index household						
Poor	62.1	66.4	68.5	51.3	53.6	58.1
Middle	14.6	12.6	13.8	15.2	13.0	13.2
Rich	23.3	21.0	17.6	33.4	33.4	28.7
Highest level of education of the mother						
No education	71.6	72.6	76.4	61.2	61.3	65.9
Primary	23.3	20.8	19.5	28.5	28.6	25.2
Secondary and higher	5.1	6.5	4.1	10.4	10.1	8.8
BMI of the mother						
Less than 18.5	65.3	66.6	65.1	64.5	62.4	65.5
18.5–24.9	6.1	5.8	6.3	5.6	6.4	6.2
>=25	28.6	27.5	28.6	29.9	31.2	28.4
Husband/partner's education level						
No education	56.0	56.1	60.1	42.1	43.7	49.9
Primary	32.7	28.5	29.3	38.5	38.9	33.2
Secondary and above	11.4	15.5	10.6	19.4	17.4	16.9
Husband/partner's occupation						
No work	9.9	11.3	10.8	9.2	9.8	11.3
Had work	90.1	88.7	89.2	90.8	90.2	88.7
Mother occupation						
No work	57.6	63.9	60.4	55.7	54.3	63.3
Had work	42.4	36.1	39.6	44.3	45.7	36.7

Moreover, the distributions of malnutrition and morbidity among under-five children in Ethiopia by region are shown in Table 2. As indicated, a high prevalence of wasting was observed in Somali (22.5%) and the lowest wasting rate was in Addis Ababa (3.1%), which shows that the Somali region needs urgent food security interventions. Stunting, indicative of chronic malnutrition, was high in the Amhara region (43.7%) and comparatively low in Addis Ababa (11.6%), indicating long-term strategies to reduce chronic form malnutrition are necessary to target regions with high rates of malnutrition. The Afar and Benishangul-Gumuz regions showed high rates of underweight (37.0% and 32.0%, respectively), showing combined impacts of acute and chronic malnutrition. Strengthening communities through agricultural training and addressing different factors causing food shortages in these regions are necessary. The highest percentage of diarrhea was obtained in Southern Nations, Nationalities, and Peoples' Region (SNNPR) (13.9%) and Gambela (13.5%), and fever prevalence was highest in Tigray (23.7%), indicating that improving water and sanitation infrastructure is vital to prevent morbidities in these regions. High prevalences of anemia were obtained in Somali (73.6%), Dire Dawa (71.9%), and Afar (70.0%), and in contrast, Addis Ababa showed a relatively low rate of anemia (47.9%), indicating iron supplementation programs and access to iron-rich foods (cereals and vegetables) in highly prevalent regions is necessary. In addition, this finding suggests that implementing strategies such as improving healthcare infrastructure, community-based interventions, fostering partnerships and collaborations, and enhancing water and sanitation can help overcome the logistical challenges in regions such as Somali and Afar, leading to better healthcare and nutrition outcomes for children.

**Table 2:** Distribution of malnutrition and morbidity among under-five children in Ethiopia by region (EDHS, 2016).

Region	Malnutrition			Morbidity		
	Wasting (%)	Stunted (%)	Underweight (%)	Diarrhea (%)	Fever (%)	Anemic (%)
Tigray	12.3	37.0	21.7	12.7	23.7	55.5
Afar	20.0	43.2	37.0	11.1	16.6	70.0
Amhara	10.1	43.7	26.9	13.2	12.4	45.7
Oromia	11.3	34.5	21.5	11.4	14.7	64.4
Somali	22.5	27.2	26.2	6.6	9.0	73.6
Benishangul	10.5	41.1	32.0	9.5	7.6	44.9
SNNPR	6.7	37.2	20.5	13.9	14.5	52.8
Gambela	15.2	24.7	18.6	13.5	15.4	58.0
Harari	11.0	31.7	18.5	11.2	10.6	67.4
Addis Ababa	3.1	11.6	3.6	8.5	14.9	47.9
Dire Dawa	10.7	38.0	24.7	12.6	12.6	71.9

### 3.2 Measurement model

Confirmatory factor analysis (CFA) was utilized to evaluate the measurement model. During this process, the factor loadings for each item within each construct were checked, and those with low factor loadings were excluded. The measurement model's fitness was evaluated using two thresholds: CFI: ≥0.9 and SRM: <0.08. The composite reliability test was used to assess construct dependability at a threshold of ≥0.7. The constructs’ validity was tested using the AVE with a threshold of ≥0.5. As indicated in Table 3, all the values met their respective acceptance thresholds, indicating that the constructs were reliable and valid, and the measurement model adequately fit the data. The chi-square test of model fitness was significant as expected, due to its sensitivity to sample size during model testing.

**Table 3:** Reliability and validity tests of constructs and model fit statistics for the measurement model (EDHS, 2016).

Constructs/latent variables	No. indicators	CR	AVE	CFI	SRMR	$\chi^{2}$ (*P*-value)
Malnutrition	3	0.934	0.896	0.99	0.001	198.77 (0.00)
Morbidity	3	0.792	0.547	0.99	0.01	79.23 (0.001)
Socioeconomic conditions	6	0.802	0.861	0.98	0.06	89.45 (0.001)

CR, composite reliability; AVE, average variance extracted; RMSEA, root mean square error of approximation; CFI, comparative fit index.

### 3.3 Structural model

The fitness of the structural model was assessed using various metrics as indicated in Table 4. After iteratively modifying the model until all indices fulfilled the criteria for a decent fit, the root mean square error of approximation (RMSEA) returned a value of 0.03, falling within the acceptable range of <0.08. The CFI yielded a value of 0.98, which was within the permitted range of >0.9. The TLI was computed at 0.92, slightly higher than the allowed range of >0.90. The SRMR value was 0.057, which fell within the permitted range of <0.08. NNFI returned a result of 0.99, which was within the allowed range of >0.90. The chi-square divided by the degrees of freedom yielded a value of 4.16, which fell within the acceptable range of 3–5. All of the indices fell within an acceptable range, showing that the model was capable of predicting the data (Table 4).

**Table 4:** Model fitness indices for the modified structural model (EDHS, 2016).

Metric	RMSEA	CFI	TLI	SRMR	NNFI	$\chi^{2}$/df
Structural model	0.03	0.98	0.92	0.057	0.99	4.16
Acceptable range	<0.08	>0.9	>0.90	<0.08	>0.90	3–5

RMSEA, root mean square error of approximation; CFI, comparative fit index; TLI, Tucker–Lewis index; SRMR, standardized root mean square residual; NNFI, non-normed fit index.

#### 3.3.1 Direct effects in the structural model

Table 5 provides insights into the direct effects within the model, indicating that time to get water in minutes with categories 6–16 and ≥30 min, preceding birth interval, type of toilet facility, wealth index household, child is a twin, place of delivery, size of child at birth, number of child under 5 years in household, and socioeconomic condition had significant direct effects on child's malnutrition. Preceding birth interval of 49 and above months, rich household, place of delivery, size of child at birth, number of children under 5 years in the household, and socioeconomic condition had direct significant effects on child morbidity.

**Table 5:** Direct effects of risk factors on child malnutrition and morbidity (EDHS, 2016).

Risk factor	Malnutrition $\hat{\beta }$ (95% CI)	*P*-value	Morbidity $\hat{\beta }$ (95% CI)	*P*-value
Time to get water in min (ref <5)				
Time to get water in min (6–16)	0.022 (−0.013 to 0.057)	0.216	0.026 ( −0.068 to 3.017)	0.238
Time to get water in min (16–30)	0.047 (0.009 to 0.086)	0.016	0.017(−0.064 to 5.030)	0.478
Time to get water in min (30 and above)	0.077 (0.034 to 0.120)	0.000	0.006 (−0.058 to 0.046)	0.823
Birth order of child (ref. first)				
Second	−0.004 (−0.031 to 0.022)	0.755	0.003 (−0.030 to 0.035)	0.871
Third or later	0.018 (−0.011 to 0.046)	0.219	0.022 (−0.013 to 0.057)	0.213
Preceding birth interval (ref. less than 24 months)				
25–48 months	−0.045 (−0.072 to −0.017)	0.001	−0.007 (−0.041 to 1.027)	0.681
49 months and above	−0.118 ( −0.150 to −0.087)	0.000	−0.039 (−0.00001 to 1.078)	0.050
Source of drinking water (ref. piped water)				
Public tap	0.013 (−0.012 to 0.037)	0.310	−0.017 (−0.013 to 0.047)	0.276
Protected spring	−0.022 (−0.047 to 0.002)	0.077	−0.004 (−0.026 to 0.034)	0.793
Type of toilet facility (ref. flushing toilet)				
Latrine	0.100 (0.043 to 0 0.157)	0.001	0.007 (−0.062 to 0.076)	0.851
No facility	0.108 (0.048 to 0.168)	0.000	0.012 (−0.060 to 0.084)	0.739
Wealth index household (ref. poor)				
Middle	−0.040 (−0.064 to −0.017)	0.001	−0.011 (0.017 to 0.999)	0.045
Rich	−0.100 (−0.133 to −0.068)	0.000	−0.042 (0.002 to 0.981)	0.039
Child is a twin (ref. single)				
Multiple	0.038 (0.017 to 0.059)	0.000	0.005 (−0.030 to 0.022)	0.729
Place of delivery (ref. home)				
Health facility	−0.047 (−0.073 to −0.022)	0.000	−0.074 (0.043 to 0.985)	0.000
Size of child at birth (ref. small)				
Average	−0.064 ( −0.089 to −0.039)	0.000	0.091 (−0.122 to −0.060)	0.000
Large	−0.104 ( −0.129 to −0.078)	0.000	0.057 (−0.089 to −0.026)	0.000
Body mass index of child (less than 18.5)				
18.5–24.9	0.002 ( −0.019 to 0.023)	0.823	0.000 (−0.026 to 0.026)	0.985
≥25	−0.000 (−0.021 to 0.021)	0.984	0.016 (−0.009 to 0.042)	0.219
Number of children under 5 in household (below 1)				
2–3	−0.031 ( −0.057 to −0.005)	0.019	0.042 (1.004 to 2.009)	0.011
4 and above	−0.034 ( −0.057 to −0.012)	0.003	0.031 (1.011 to 2.025)	0.003
Socioeconomic condition	−0.042 (−0.076 to −0.007)	0.017	0.085 (−0.127 to −0.043)	0.000

Among a total of 8,560 children, 12.80% had wasting, 34.75% had stunting, 23.91% were underweight, and 34% had fever. The odds of malnutrition were 1.048 times (OR = 1.048, 95% CI = 0.009–0.086, *P* = 0.016) more common for children whose mothers spend 16–30 min fetching water and 1.080 times (OR = 1.080, 95% CI = 0.034–0.120, *P* = 0.000) more common for children whose mother spend 30 min and above to fetching water compared to those who spend five minutes and less, indicating longer durations significantly increase the odds of malnutrition, particularly for those spending 16–30 and 30 min and more fetching water. The odds of malnutrition were less common among children whose preceding birth interval was between 25 and 48 months (OR = 0.956, 95% CI = −0.072 to −0.017, *P* = 0.001) compared to those with a birth interval less than 24 months and the odds of malnutrition was less common among those children whose preceding birth interval was 49 months and more (OR = 0.889, 95% CI = −0.150 to −0.087, *P* = 0.000) compared to those in the reference category, indicating that longer preceding birth intervals (25–48,and 49 months and above) are associated with significant decreases in the odds of malnutrition. The odds of malnutrition were higher among children who use latrine facilities (OR = 1.105, 95% CI = 0.043–0.157, *P* = 0.001) and those without a toilet (OR = 1.114, 95% CI = 0.048–0.168, *P* = 0.000) facilities compared to those who use a flushing toilet, indicating that using a latrine significantly increases the odds of malnutrition in children, with an increase of 10.5%. Similarly, lacking toilet facilities was associated with an 11.4% increase in malnutrition odds, which was also statistically significant. The odds of malnutrition were lower among children from middle-income (OR = 0.961, 95% CI = −0.064 to −0.017, *P* = 0.001) and rich households (OR = 0.905, 95% CI = −0.113 to −0.068, *P* = 0.000) compared to those from poor households, indicating that children from middle-income households had a 3.9% decrease in the odds of child malnutrition, which was significant. Similarly, being from a rich household was associated with a 9.5% decrease in malnutrition odds, which was also significant. The odds of malnutrition were higher among children who were a multiple (OR = 1.038, 95% CI = 0.017–0.059, *P* = 0.000) compared to children who were not a multiple, indicating being a multiple (twins or more) was significantly associated with an increased odds of child malnutrition, reflecting a 3.8% rise in odds, emphasizing potential health risks for multiples. The odds of malnutrition were lower for children delivered in a health facility (OR = 0.954, 95% CI = −0.073 to −0.022, *P* = 0.000) compared to those who were delivered home, indicating that delivering in a health facility was associated with a 4.6% reduction in the odds of child malnutrition, highlighting the protective benefits of facility-based deliveries. The odds of malnutrition of the child were lower among children categorized as average size (OR = 0.938, 95% CI = −0.089 to −0.039, *P* = 0.000) or large size (OR = 0.901, 95% CI = −0.129 to −0.078, *P* = 0.000) as compared to children categorized as small size, indicating being of average size was linked to a 6.2% decrease in malnutrition odds, while large children had a 9.9% decrease in odds. The odds of malnutrition were lower among households with two to three children (OR = 0.969, 95% CI = −0.057 to −0.005, *P* = 0.019) and four and above children (OR = 0.966, 95% CI = −0.057 to −0.012, *P* = 0.003) compared to households with one child, indicating that having two to three children and four or more children was associated with a significant reduction in the odds of child malnutrition (Table 5).

The odds of morbidity were lower among children from middle-income (OR = 0.989, 95% CI = 0.017–0.999, *P* = 0.045) and rich households (OR = 0.959, 95% CI = 0.002–0.981, *P* = 0.039) compared to those from poor households, indicating that children from middle-income households were associated with a 1.1% decrease in childhood morbidity odds, which was significant. Similarly, being from a rich household was associated with a 4.1% decrease in morbidity odds, which was significant. The odds of morbidity were lower for children delivered in a health facility (OR = 0.928, 95% CI = 0.043–0.985, *P* = 0.000) compared to those who were delivered at home, indicating that delivering in a health facility was associated with a 7.2% reduction in the odds of child morbidity, highlighting the protective benefits of facility-based deliveries. The odds of morbidity of the child were higher among children categorized as average size (OR = 1.095, 95% CI = −0.122 to −0.060, *P* = 0.000) or large size (OR = 1.058, 95% CI = −0.089 to −0.026, *P* = 0.000) compared to children categorized as small size, indicating that being of average size was linked to a 6.2% decrease in morbidity odds, while large children had a 9.9% decrease in odds. The odds of morbidity were higher among those households with two to three children (OR = 1.043, 95% CI = 1.004–2.009, *P* = 0.011) and four and above children (OR = 1.031, 95% CI = 1.011–2.025, *P* = 0.003) compared to households with one child, indicating that having two to three children and four or more children was associated with a significant increase in the odds of child morbidity. Socioeconomic conditions had a significant impact on child morbidity (OR = 1.088, 95% CI = −0.127 to −.043, *P* = 0.000) (Table 5).

**Figure 3:**
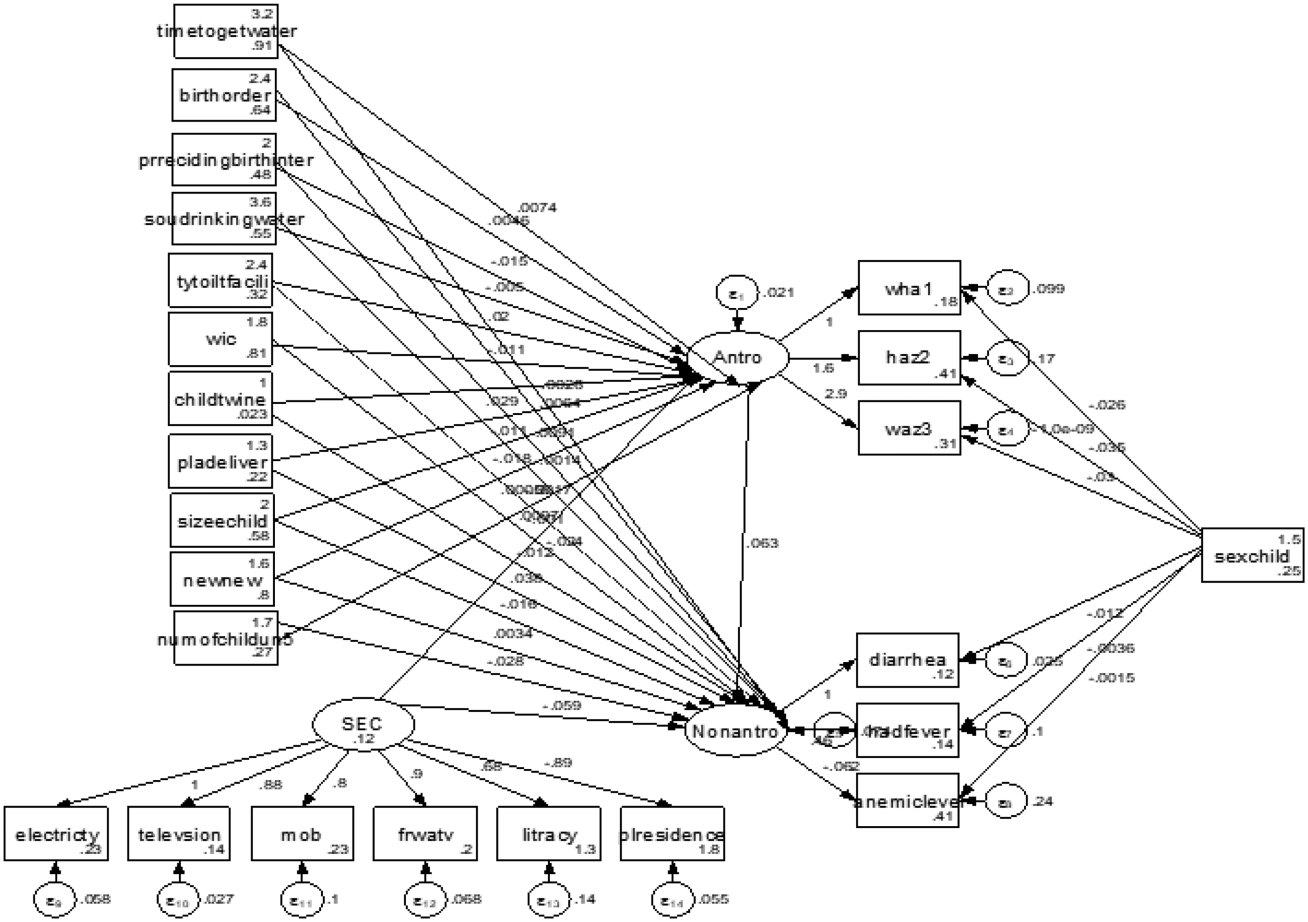
Structural equation model of effects of selected covariates on the latent variables, malnutrition and morbidity, and observed indicators.

The structural model in Figure 3 depicts the finalized structural model after re-specification of the conceptual model, which includes revisions based on modification indices to improve the model's fit.

#### 3.3.2 Indirect and total effects in the mediation analysis of the structural model

Table 6 shows the indirect and total effects of risk factors on child morbidity via malnutrition. The estimation results for indirect effects indicated that time to get water, preceding birth interval, type of toilet facility, wealth index of household, child is a twin, place of delivery, size of child, and number of children exhibit an indirect effect on morbidity through malnutrition. For instance, the result revealed that children in households with 6–16 min (β = 0.001, 95% CI = 0.001–0.002, *P* = 0.039), 16–30 min (β = 0.001, 95% CI = 0.001–0.003, *P* = 0.049), and 30 min and above (β = 0.002, 95% CI = 0.001–0.003, *P* = 0.028) water access were more likely to be exposed to diseases than children in households with 5-min water access and less via malnutrition and they had a significant total effect on morbidity. A preceding birth interval of 25–48 months (β = −0.001, 95% CI = −0.002 to −0.001, *P* = 0.035) and 49 and above months (β = −0.003, 95% CI = −0.005 to −0.001, *P* = 0.009) showed a slight and strong reduction in child morbidity via malnutrition, respectively, compared to children with short birth intervals via malnutrition, and they had a significant total effect on morbidity. Households in the middle-income category (β = −0.001, 95% CI = −0.002 to −0.001, *P* = 0.031) and in the rich category (β = −0.002, 95% CI = −0.004 to −0.001, *P* = 0.011) showed reduced child morbidity via the malnutrition pathway. Children who were delivered in a health facility (β = −0.001, 95% CI = −0.002 to −0.001, *P* = 0.027) showed a slight reduction in morbidity through malnutrition as compared with being delivered at home. Time to get water in min, place of delivery, size of child, and number of children under 5 years had significant indirect and total effects on morbidity through malnutrition and socioeconomic conditions had a significant total effect on morbidity via malnutrition. Figure 2 indicates that the sex of the child significantly determined the stunting, wasting, underweight, diarrhea, anemia, and fever status of under-five children. A female child was less likely to be stunted and wasted than a male child (OR = 0.49. 95% CI = 0.28–0.99 and OR = 0.96, 95% CI = 0.39–0.99). A female child was less likely to have diarrhea and fever compared to a male child (OR = 0.98, 95%CI = 0.39–0.99 and OR = 0.99, 95% CI = 0.29–0.99) and more likely to have anemia (OR = 1.002, 95% CI = 0.39–2.71).

**Table 6:** Indirect and total effects of risk factors on morbidity via malnutrition (EDHS, 2016).

Path via malnutrition	Indirect effect $\hat{\beta }$ (95% CI)	*P*-value	Total effect $\hat{\beta }$ (95% CI)	*P*-value
Time to get water in min (6–16) → morbidity	0.001 (0.001 to 0.002)*	0.039	−0.0173 (−0.0471 to 0.0125)	0.051*
Time to get water in min (16–30) → morbidity	0.001 (−0.001 to 0.002)*	0.049	−0.0091 (−0.0375 to 0.0191)	0.026*
Time to get water (30 and above) → morbidity	0.002 (0.001 to 0.003)*	0.028	−0.0016 (−0.0285 to 0.0253)	0.009*
Birth order: second → morbidity	−0.001 (−0.001 to 0.001)	0.757	0.0017 (−0.0207 to 0.0242)	0.879
Birth order: third or later →morbidity	−0.001 (−0.001 to 0.001)	0.261	0.0121 (−0.0064 to −0.0307)	0.199
Preceding birth interval: (25–48) → morbidity	−0.001 (−0.002 to −0.001)*	0.035	−0.0045 (−0.0219 to −0.0129)	0.611
Preceding birth interval: (49 and above) → morbidity	−0.003 (−0.005 to −0.001)*	0.009	0.0203 (−0.0026 to −0.0433)	0.082
Drinking water: public tap → morbidity	0.001 (−0.001 to 0.001)	0.340	0.0165 (−0.0124 to −0.0453)	0.263
Drinking water: protected spring → morbidity	−0.002 (−0.001 to −0.001)	0.135	0.0019 (−0.0165 to −0.0205)	0.835
toilet facility: latrine → morbidity	0.002 (0.001 to 0.004)*	0.030	0.0054 (−0.0303 to −0.0410)	0.768
toilet facility: no facility → morbidity	0.002 (0.001 to 0.004)*	0.029	0.0085 (−0.0292 to −0.0462)	0.658
Wealth index: middle → morbidity	−0.001 (−.002 to −0.001)*	0.031	0.0071 (−0.0140 to −0.0283)	0.509
Wealth index: rich → morbidity	−0.002 (−0.004 to −0.001)*	0.011	0.0211 (−0.0009 to −0.0432)	0.061
Child is a twin: multiple →morbidity	0.002 (0.001 to 0.005)*	0.028	−0.0053 (−0.0492 to −0.0385)	0.811
Place of delivery: health facility → morbidity	−0.001 (−0.002 to −0.001)*	0.027	0.0400 (0.0226 to −0.0574)	0.000*
Size of child: average → morbidity	−0.001 (−0.002 to −0.001)*	0.015	−0.0489 (−0.0649 to −0.0327)	0.000*
Size of child: large → morbidity	−0.002 (−0.004 to −0.001)*	0.008	−0.0346 (−0.0522 to −0.0171)	0.000*
Body mass index: (18.5–24.9) → morbidity	0.001 (−0.001 to −0.001)	0.823	−0.0002 (−0.0281 to −0.0277)	0.991
Body mass index: ≥25 →morbidity	0.00001 (−0.0004 to 0.0004)	0.984	0.0092 (−0.0055 to −0.0239)	0.219
Number of children: (2–3) → morbidity	−0.0006 (−0.0013 to 0.0001)*	0.032	−0.0230 (−0.0402 to −0.0059)	0.009*
Number of children: (4 and above) → morbidity	−0.002 (−0.0039 to −0.0001)*	0.040	−0.0401 (−0.0831 to −0.0028)	0.067
SEM	−0.0012 (−0.0025 to 0.0001)	0.068	−0.0653 (−0.0973 to −0.0334)	0.000*

*Indicates a significance at 5%.

## 4 Discussion

Using data from the 2016 Ethiopia Demographic and Health Survey, we fitted a structural equation model to the morbidity (diarrhea, fever, and anemia), malnutrition (stunting, wasting, and underweight), and socioeconomic conditions of households (literacy, place of residence, access to electricity, television, mobile, and frequency of watching television). The structural equation models provided the latent effects on childhood malnutrition and morbidity within a combined modeling framework. Furthermore, structural equation modeling enabled the investigation of malnutrition as a mediator of the relationship between chosen risk factors and latent variable morbidity.

Malnutrition remains a significant health issue for Ethiopian children under the age of 5, with our study revealing high rates of stunting (34.75%), wasting (12.08%), underweight (23.91%), fever (13.9%), diarrhea (11.2%), and anemia (59.7%) among 8,592 under-five children, which remain high in comparison to Ethiopia's Seqota Declaration, which aims to end child malnutrition by 2030 (28). More recent findings are consistent with this, with minor variances due to methodological discrepancies (29). The high prevalence of childhood malnutrition and morbidity highlights the urgent need to enhance maternal and child healthcare in Ethiopia. Our findings suggest that improving access to healthcare and strengthening the availability of water are critical to reducing childhood diseases and enhancing overall child health outcomes. These actions are key to advancing Sustainable Development Goal (SDG) 3 by ensuring healthy lives and promoting wellbeing for all. Furthermore, our results indicate that achieving the SDGs related to nutrition, health, and sanitation in Ethiopia by 2030 will require multi-sectoral approaches, including enhancing healthcare access, expanding education, and addressing socioeconomic inequalities. Focused interventions targeting rural populations, where child malnutrition and morbidity are most prevalent, will be essential to accelerate progress toward these goals. A study conducted in South Ari District, southern Ethiopia, showed the prevalence of wasting among children aged 6–59 months was 9.10% (31) and a prevalence of 8% was found in Kenya (32). The 2019 Ethiopia Mini Demographic Health Survey (EMDHS) finding for the southern region was 6.3% (33) which was a lower prevalence rate compared with our findings (12.08%). However, our study showed a lower prevalence of wasting compared to results from Bangladesh (18.2%) (34). These discrepancies might be explained partly due to socioeconomic differences, seasonal variation, feeding habits of the study population, and sample size and study setting differences. Our study revealed that the prevalence of stunting and chronic malnutrition was 34.75%. This finding was lower than studies in South Ari District, southern Ethiopia (59.97%) (31), different parts of Ethiopia (42.3%–64.5%) (32–39), and rural Bangladesh (36.8%) (31). Possible explanations for this difference might be variations in the socioeconomic status, sample size, study setting, cultural factors, and feeding habits of the study population.

Furthermore, our findings revealed that anemia affects a significant percentage of under-five children, estimated to be approximately 59.73% nationwide. Almost comparable results were reported in Woldekidan et al. (40), which revealed that the prevalence of anemia among under-five children in Ethiopia was 62.0%, highlighting widespread dietary shortages that can have a negative impact on cognitive and physical development. Our findings showed that diarrhea (11.24%), fever (13.93%), and anemia (59.73%) were prevalent issues, which are commonly linked to malnutrition and environmental factors, contradicting Ethiopia's SDGs to end preventable deaths of newborns and children under the age of 5 by 2030 (41). These findings indicate that malnutrition-related measures, such as improving access to healthcare, sanitation, and nutritious food, are crucial for lowering morbidity and malnutrition among Ethiopia's under-five children.

This study showed that the prevalence of wasting was high in the Somali region (22.5%) and low in Addis Ababa (3.1%), stunting was higher in the Amhara region (43.7%) and lower in Addis Ababa (11.6%), underweight was higher in the Afar (37.0%) and Benishangul-Gumuz regions (32.0%) and lower in Addis Ababa (3.6%), indicating regional disparities in childhood nutrition across Ethiopia, and these disparities may be largely influenced by socioeconomic, environmental, and health infrastructure variations between regions. The highest percentage of diarrhea was found in SNNPR (13.9%) and Gambela (13.5%), and our result was inconsistent with a previous meta-analysis study that found smaller proportions of diarrhea in the regions (42). Fever prevalence was highest in Tigray (23.7%), and high percentages of anemia were obtained in Somali (73.6%), Dire Dawa (71.9%), and Afar (70.0%), while Addis Ababa had a relatively low rate of anemia (47.9%). This study recommended investing in clean water supply and sanitation facilities, educating communities on safe water storage, strengthening disease surveillance, encouraging community health education on recognizing early symptoms and seeking prompt treatment, and distributing iron-folic acid and vitamin supplements to areas with high rates of morbidity and malnutrition.

This study also showed that children living in rural areas had a higher frequency of malnutrition and morbidity than children living in cities. These findings were consistent with research conducted in Bangladesh (43, 44). Children living in rural areas were stunted (88.4%), wasted (85.6%), and underweight (90.3%), with higher rates than in a previous study (42), which found that the prevalence of malnutrition among children in rural Ethiopia was 48.5% using the 2014 EMDHS. Rural children were more vulnerable to these illnesses, possibly due to restricted access to healthcare, clean water, and a balanced diet in the rural areas of Ethiopia.

This study found that the prevalence of diarrhea in the rural areas of Ethiopia was 82.8%, which was greater than the pooled prevalence of diarrhea in Ethiopia, which was 22% (45). This study found that anemia prevalence was 83.0% among children living in the rural region of Ethiopia, which was higher than in Alebel et al. (45), which showed that 46.6% of children living in rural were anemic. This prevalence (83.0%) was higher than research conducted in Egypt (39%) (46), the People's Republic of China (13.4%) (47), Serbia (7.7%) (48), and Korea (8.4%) (49). This discrepancy could be attributed to differences in socioeconomic status, education level, and nutritional intake. Anemia prevalence was 17% among urban inhabitants in this study, and nearly the same result was reached in Fantay Gebru et al. (50), which also showed a 16% incidence for urban inhabitants. Similar to the findings of the 2011 Ethiopian demographic and health census, 31% of respondents were rural, whereas 16% were urban. Our study showed that across all health indicators, i.e., stunting, wasting, underweight, diarrhea, fever, and anemia, children in rural areas were disproportionately affected compared to their urban counterparts. These results underscore the disparity in health and nutritional status between urban and rural areas in Ethiopia, indicating that a multi-pronged approach to nutrition, healthcare, and infrastructure in rural areas is critical.

The study found that high percentages of stunted (50.9%), wasting (49.7%), and underweight children (51.0%) were born to mothers aged 15–19 years. Evidence from 55 low- and middle-income countries, including those in sub-Saharan Africa, supports our findings (51–53). Children with younger mothers are at a higher risk of malnutrition, perhaps because younger mothers are less likely to have completed their education and frequently lack proper prenatal and postnatal care. This, in turn, impacts their ability to provide sufficient nourishment and healthcare for their children. Our findings show that young maternal age, specifically giving birth between the ages of 15 and 19 years, is related to a higher risk of negative child health outcomes such as diarrhea (50.2%), fever (50.1%), and anemia (49.2%). These findings were consistent with previous investigations (54–56). Maternal health knowledge, a lack of access to healthcare, and economic factors common among younger mothers may all contribute to these findings. Addressing these health challenges requires targeted interventions in maternal education, healthcare access, and nutritional programs, particularly for young mothers, to help reduce the prevalence of anemia, diarrhea, and febrile illnesses among children in these demographics.

The study found that children in male-headed households had higher rates of malnutrition and morbidity, including stunting (79.7%), wasting (76.5%), and underweight (76.5%), and illnesses such as diarrhea (81.8%), fever (78.5%), and anemia (78.8%), than those in female-headed households. Our findings were validated by other studies (57, 58). This could be due to differences in caring techniques, resource allocation, or health-seeking behavior that improve children’s health outcomes. Our study found that children who were breastfed within the first hour of birth had lower rates of stunting (5.0%), wasting (4.8%), underweight (5.0%), diarrhea (4.7%), fever (4.1%), and anemia (4.9%) than those who were breastfed later, indicating that early breastfeeding provides critical nutrients and antibodies that support the immune system. Delayed breastfeeding, however, was associated with an increased risk of these health concerns. Our findings were supported by systematic reviews and meta-analyses (59, 60), and a study conducted in Germany (61). Our findings suggest that health services should stress the benefits of breastfeeding right after birth.

This study found that children of mothers with no formal education had higher rates of malnutrition, including stunting (71.6%), wasting (72.6%), underweight (76.4%), and higher morbidity rates from conditions such as diarrhea (61.2%), fever (61.3%), and anemia (65.9%), indicating that maternal education is strongly associated with better child health outcomes. A study conducted in 27 countries supports our findings (62). The research indicated that the level of education of mothers significantly affects the nutritional status of the child and morbidity, where malnutrition and morbidity decrease as the level of education of the mother increases. This finding is in line with previous studies (63, 64). This shows that an educated mother is more likely to learn proper feeding practices, improve hygiene, and gain improved access to knowledge and awareness.

The study showed that children of working mothers are less likely to be malnourished and diseased as compared to children of non-working mothers. The finding was consistent with some previous studies (65, 66). In this study, children being breastfed were more likely to be malnourished and diseased as compared to non-breastfed children. This result was inconsistent with previous findings (67–69). The study result indicates that malnutrition and morbidity in these groups may be influenced by factors beyond breastfeeding, such as maternal nutrition, household socioeconomic conditions, or long-term food security. In this study, children without anemia were less likely to be malnourished compared to anemic children. Some studies found similar results (63, 70). Our study showed that the odds of malnutrition and morbidity were lower among children whose preceding birth interval was between 25 and 48 months compared to those with a birth interval less than 24 months and the odds of malnutrition and morbidity were lower among those children whose preceding birth interval was 49 months and more compared to those in the reference category, indicating that birth intervals are beneficial for reducing the risk of malnutrition and related health issues in children. This protective impact could be attributed to increased maternal recovery and resource allocation, providing each child with superior nutritional and health outcomes. A similar result was obtained previously (71, 72). Our results suggest a marginally higher prevalence of malnutrition- and morbidity-related indicators among male children compared to female children. These differences could be influenced by several factors, including biological differences in metabolism and immune responses and potential gender-based variations in care practices. This result was consistent with previous findings (63, 73). The odds of malnutrition and morbidity were lower for children born second compared to those born first. The odds of malnutrition were higher among those children born third or later compared to those born first. Neither being born second nor being born third or later showed statistically significant associations with malnutrition. Similar results were shown in Takele et al. and Lamberti et al. (65, 69).

The odds of malnutrition and morbidity were higher among children who used latrine facilities and in those without toilet facilities compared to those who used flushing toilets, indicating that using a latrine significantly increases the odds of malnutrition and morbidity in children, with an increase of 10.5%. Similarly, lacking toilet facilities was associated with an 11.4% increase in malnutrition odds, which was also statistically significant. This finding conforms with (65, 66).

Our results revealed that children from households with 6–16, 16–30, and 30 min and above water access were more likely to be exposed to diseases than children with households with 5-min water access and less via malnutrition and they had a significant total effect on morbidity, indicating that addressing water accessibility issues and ensuring closer, consistent access to safe water are crucial for improving childhood health and malnutrition. A study that supports our results showed that the availability and accessibility of clean water significantly impact childhood health (72). A preceding birth interval of 25–48 and 49 months and above were shown to have a slight and strong reduction in child morbidity via malnutrition, respectively, compared to children with a short birth interval via malnutrition, but they had an insignificant total effect on morbidity. Our findings indicate that educating communities about the benefits of birth intervals of at least 25–48 months or longer and emphasizing the role of optimal spacing in reducing malnutrition, which indirectly reduces child morbidity, is necessary. This study found that a child's birth size had a significant direct effect on malnutrition, aligning with findings from a study in India (74), which suggests that interventions should prioritize enhancing maternal nutrition and prenatal care to support healthy fetal growth. Our results indicated that rural children had higher rates of malnutrition and morbidity compared to their urban counterparts, consistent with findings from a study conducted in Ethiopia (75) and research in low- and middle-income countries (76).

The study results showed that time to get water, preceding birth interval, type of toilet facility, wealth index of household, child is a twin, place of delivery, size of child, and number of children exhibit an indirect effect on morbidity through malnutrition. Time to get water in min, place of delivery, size of child, and number of children under 5 years had a significant indirect and total effect on morbidity through malnutrition and socioeconomic conditions had a significant total effect on morbidity via malnutrition. Households in the middle-income category and those in the rich category showed reduced child morbidity via the malnutrition pathway. Children who were delivered in a health facility showed a slight reduction in morbidity through malnutrition compared with those delivered at home. This finding conforms with Takele et al. (65) and our study recommends raising awareness and encouraging facility-based deliveries, which provide improved sanitation, skilled delivery attendance, and immediate access to newborn care.

## 5 Strengths and limitations of the study

The SEM fit the data well and revealed the complex interrelationships between regional, child demographic, household, and environmental factors, as well as their direct or indirect relationship to childhood malnutrition and illness. Furthermore, the current study used a large sample size. However, the DHS data are cross-sectional, therefore they are insufficient for understanding changes in malnutrition and morbidity over time. As a result, future research should use a large number of DHS datasets to simulate a longitudinal study and investigate the stability of the important risk factors identified in this study over time.

## 6 Conclusion

The prevalence of malnutrition and illness among under-five children in Ethiopia was high when compared to other studies, indicating that childhood malnutrition and morbidity in Ethiopia remains a major health issue that must be addressed urgently. Using a structural equation model, time to get water in min (6–16 and 30 min and above), preceding birth interval, type of toilet facility, wealth index household, child is a twin, place of delivery, size of child at birth, number of children under 5 in the household, and socioeconomic condition had a significantly direct effect on childhood malnutrition. A preceding birth interval of 49 months and above, a rich household, place of delivery, size of child at birth, number of children under five in the household, and socioeconomic condition had statistically direct significant effects on child morbidity. Time to get water, preceding birth interval, type of toilet facility, wealth index of household, child is a twin, place of delivery, size of child, and number of children exhibited an indirect effect on morbidity through malnutrition. Time to get water in min, place of delivery, size of child, and number of under-five children had significant indirect and total effects on morbidity through malnutrition and socioeconomic conditions had a significant total effect on morbidity via malnutrition. Policymakers must consider the impact of these key elements when developing policies to improve the health of Ethiopian children under the age of 5 years. This study also suggests that improving mothers’ working conditions will enhance their economic status and, as a result, fulfill their children's basic needs. The Ethiopian government urgently needs to establish initiatives targeting the regions of Afar, Dire Dawa, Gambela, Harari, and Somali to develop strategies to improve the nutritional condition and health of Ethiopia's under-five children.

## Data Availability

Publicly available datasets were analyzed in this study. The data can be found here: https://www.dhsprogram.com/Data/.
